# How does the British Soft Drink Association respond to media research reporting on the health consequences of sugary drinks?

**DOI:** 10.1186/s12992-021-00719-y

**Published:** 2021-07-02

**Authors:** Marco Zenone, Diego Silva, Julia Smith, Kelley Lee

**Affiliations:** 1 Faculty of Public Health & Policy, London School of Hygiene and Tropical Medicine, Keppel Street, London, WC1E 7HT UK; 2 Faculty of Health Sciences, Simon Fraser University, 8888 University Drive, Burnaby, British Columbia V5A 1S6 Canada; 3 Sydney Health Ethics, School of Public Health, The University of Sydney, Edward Ford Building, A27 Fisher Rd, University of Sydney, Sydney, NSW 2006 Australia

**Keywords:** Sugar-sweetened beverages, Sugary drinks, Evidence, Public health, Commercial determinants of health, Precautionary

## Abstract

**Background:**

Sugar-sweetened beverages (SSBs) are the leading global source of added sugar intake and their consumption is associated with negative health outcomes, such as diabetes, cancers, cardiovascular diseases, and overall mortality. Despite consensus within the public health community about the need to reduce sugar intake, the non-alcoholic beverage industry engages in efforts to publicly undermine the evidence base surrounding the harmful effects of SSBs. There has been limited investigation of how SSB industry actors engage in public debates to challenge public health research and policy on SSBs. To address this gap, we thematically analyze the public comments and press releases of the British Soft Drinks Association (BSDA) since May 2014.

**Results:**

A total of 175 news articles and 7 press releases were identified where the BSDA commented upon new SSB research in public settings. In these comments, four strategies were observed to undermine new research. First, the BSDA challenged study rigour and research design (*n* = 150). They challenged the policy implications of research by stating observational studies do not demonstrate causation, refuted data sources, questioned researcher motivations, and claimed research design did not account for confounding factors. Second, the BSDA positioned themselves as an altruistic public health partner (*n* = 52) intent on improving population-level nutrition citing their voluntary industry commitments. Third, the BSDA promoted concepts of safety that align with industry interests (*n* = 47). Lastly, the BSDA argued that the lifestyle of individual consumers should be the focus of public health interventions rather than the industry (*n* = 61).

**Conclusion:**

The findings illustrate the BSDA reliance on arguments of causation to discredit research and avoid policy interventions. Given the attention by the BSDA regarding the purported lack of evidence of causation between SSBs and non-communicable diseases, it is imperative that members of the public health community try to educate policy makers about (a) the complex nature of causation; (b) that evidence in favour of public health interventions cannot, and do not, solely rely on causation studies; and (c) that public health must sometimes abide by the precautionary principle in instituting interventions.

## Introduction

Non-communicable diseases (NCDs) were responsible for 41 million deaths in 2017 or 71% of all deaths globally [1]. There is now clear evidence that a significant contributor to the risk of developing NCDs is an excess intake of added sugars [2]. According to the American Heart Association, excess added or free sugar intake amounts to 37.5 g/9 teaspoons for adult males and 25 g/6 teaspoons for adult females daily [3]. The leading source of added sugar intake comes from sugar-sweetened beverages (SSBs) [4, 5]. Increased consumption of SSBs is thus associated with weight gain, obesity, and an overall increase in risk of NCDs such as diabetes and various forms of cancer [6–11]. While these trends were previously observed in high-income countries primarily, alarming increases in NCDs associated with excess sugar intake is now occurring in low- and middle-income countries [12]. In response, the World Health Organization (WHO) has called for action to reduce the consumption of SSBs [13]. Among the varied measures being adopted, governments worldwide have introduced increased taxation of SSBs to raise their price and discourage consumption [14].

Contrary to broad consensus within the public health community about the need to reduce excess sugar consumption, it is now well-documented that the food industry has engaged in longstanding efforts to undermine and challenge the existing evidence base [15–19]. From the 1960s onwards, the sugar industry recruited scientists to downplay the role of sugar as a contributing factor to heart disease, shifting public concerns to the need to reduce consumption of fats [15, 20, 21]. The International Life Sciences Institute (ILSI), founded in 1978 by a former Coca-Cola executive and financed by companies such as Coca-Cola, Nestlé, McDonald’s and PepsiCo, describes itself as a “non-profit, worldwide organization whose mission is to provide science that improves human health and well-being and safeguards the environment.” [22] Its activities have included funding research, and its dissemination worldwide, attributing NCDs to consumer choices, ageing, and lack of physical activity rather than the production and consumption of specific food products [23]. In China, for example, ILSI position statements and recommendations were incorporated into government guidelines to combat the country’s rapid rise in obesity rates since the early 2000s [24, 25]. Manufacturers of SSBs, the leading source of added sugar intake, have advanced similar themes. For example, in 2015 the Coca Cola Company’s “science-based” approach to the obesity epidemic, promoted through its non-profit Global Energy Balance Network, sought to shift public attention from reduced sugar consumption to physical activity [19]. The beverage industry as a whole has also disputed evidence demonstrating the effectiveness of public health interventions targeted at SSBs, including fiscal measures such as increased taxation. In a 2016 review of sixty studies investigating the link between SSBs and health outcomes such as obesity or diabetes, it was found that the 26 studies funded by industry found no association [26]. Despite suggestions of bias in industry-funded studies, the American Beverage Association argued that “research we fund adheres to the highest standards of integrity for scientific inquiry” [27].

Amid growing evidence of industry interference in the scientific process underpinning public health research and policy concerning excess sugar intake, and health and nutrition more broadly [28, 29], the public health community has sought to counter these activities by rebutting industry claims in the public domain. This has been in response to the industry’s strategic use of public-facing messaging – defined as communicative actions promoting a view or stance – in the form of blogs, social media and political advertising campaigns, to challenge new research perceived as threatening to industry interests [30]. The limited study to date of the strategic use of public-facing messaging, and how it influences policy-debates on SSB regulation, has focused on media coverage of messaging by public health versus industry advocates, notably in the United States (US) and United Kingdom (UK), concerning the introduction of SSB taxation [31–33]. These studies contribute important understanding of “the complexity of the network of stakeholders involved in the public debate on food policies,” and their positioning supporting or opposing regulation [34]. However, there is limited attention so far to how SSB industry actors use evidence to insert themselves into public policy debates.

We begin to address this knowledge gap by presenting a case study of public-facing messaging by the British Soft Drinks Association (BSDA) to challenge public health research and evidence on SSBs. The BSDA is the “collective voice of the UK soft drinks industry” and, as the leading trade association for non-alcoholic beverage companies, regularly comments on new research and the industry’s position on SSB-related regulation [35]. We have selected the BSDA and UK context as a critical case study because the government’s introduction of the Soft Drinks Industry Levy in 2018, equivalent to a 10% excise tax, generated sustained debate around supporting evidence [36]. This case study thematically analyses the arguments put forth by industry groups to oppose the evidence base prior to, and after implementation of, the SSB levy. As well as fuller understanding of the discursive strategies used to undermine public health research, the findings offer lessons for developing effective counter arguments to advance public health outcomes.

## Methods

We began by conducting a targeted search using the media and business information database, Nexus Uni, for responses in the media by the BSDA to research related to the regulation of SSBs. The search phrase used was: “British Soft Drinks Association” AND [research OR study]. We limited our search to the period from May 2014 to May 2020 to capture: (a) the two-year period prior to announcement by the UK government of the policy in March 2016; (b) the interim period during which the government invited industry comments (April 2016–March 2018); and (c) the period since implementation on 6 April 2018. The search yielded 1183 results. Finally, given our interest in public-facing messaging, additional filters were applied to limit results to newspapers, web-based publications, newswires, magazines and journals, aggregated news sources and news. This resulted in 1005 items. A second source of data for this analysis was media releases and position statements on the BSDA’s website collected from 2016, the earliest available on the BSDA website, to the present. This search yielded 73 items for review.

The full text of all identified media reports, and BSDA media releases and position statements, (*n* = 1078) were screened for inclusion in this analysis. To be included, the item needed to meet the following criteria: (a) topic of the item specifically related to a research study linking SSBs to an adverse health outcome; and [2] item contains a direct quotation from the BSDA commenting on research findings. In total, we identified 175 news articles and 7 media releases that contain a quote from the BSDA commenting on 25 research studies. Articles were excluded for not commenting on research related to the safety of SSBs (*n* = 881); or not containing a direct quote from the BSDA (*n* = 15). The included items were reviewed a second time for confirmation. BSDA statements directly related to SSB research were compiled in a spreadsheet for thematic analysis. Additionally, the name and abstract of the study commented upon was also recorded.

For thematic analysis, the first and third authors reviewed each statement from the BSDA and independently developed themes by asking: what type of argument is being used to oppose the research? What is the basis of the argument put forth? How does the BSDA support its own argument? After initial coding, the authors agreed on four dominant BSDA response themes: challenging scientific rigour and issues of causation versus correlation; altruistic public health partner portrayal; concepts of safety; and consumer responsibility & lifestyle (see Table 1). The second author audited 25% of statements to ensure consistency. Any coding disagreements were resolved through discussion among all authors.

**Table 1:** Summary of Themes with Illustrative Quotes

Theme	Frequency	Illustrative Quote
Challenging scientific rigour and issues of causation versus correlation	150	“‘This is a health campaign statement masquerading as an academic study as even the authors accept no definitive conclusions can be drawn about cause and effect”
Altruistic public health partner	52	“We all have a role to play in helping to tackle obesity, and we hope our actions on sugar reduction, portion size, and promotion of low and no-calorie products set an example for the wider food sector.”
Concepts of safety	47	“The World Cancer Research Fund found no evidence that 100% fruit juice is carcinogenic and maintain that a daily 150 ml glass ‘can be part of a healthy, balanced diet and a healthy lifestyle’ and contribute to the 5-a-day fruit and vegetable target.”
Consumer responsibility & lifestyle	61	“Rather than singling out a particular product, we should be encouraging people to enjoy an active lifestyle.”

## Results

### Challenging scientific rigour and issues of causation versus correlation

This review found that the BSDA responded to the majority of new research on SSBs by challenging their study rigour and research design (*n* = 150). The critique is most often made against observational studies, which find an association between SSBs and an adverse health outcome, arguing that association does not demonstrate causation. For example, a systematic review and meta-analysis by Imamura et al. found that “[h] abitual consumption of sugar sweetened beverages was associated with a greater incidence of type 2 diabetes, independently of adiposity,” amounting to around 1.8 million cases in the US and 79,000 cases in the UK over a 10 year period [37]. The study, published in the *British Medical Journal* in 2015 received widespread media coverage in the UK, US, and other countries. BSDA Director General Gavin Partington responded with the following statement: “This is a health campaign statement masquerading as an academic study as even the authors accept no definitive conclusions can be drawn about cause and effect”. Similarly, a large prospective study published in the *British Medical Journal* in 2019 found “the consumption of sugary drinks was significantly associated with the risk of overall cancer and breast cancer” [38]. The BSDA commented in response: “This study reports a possible association between higher consumption of sugary drinks and an increased risk of cancer, but does not provide evidence of cause, as the authors readily admit”. The terminology that focuses on the relationship between cause and effect, and the inability of researchers to prove direct causation, is featured in the majority of BSDA statements identified.

Additional critiques of research design include refuting the source of data, findings interpretation, or noting that the study disagrees with previous industry-friendly studies. For example, in response to a 2015 study published in the journal *Circulation* that found SSBs were responsible for approximately 184,000 deaths in 2010, the BSDA challenged the study by commenting: “The researchers provide no evidence when they illogically and wrongly take beverage intake calculations from around the globe and allege that those beverages are the cause of deaths which the authors themselves acknowledge are due to chronic disease”.

A related argument used by the BSDA is to challenge studies of the adverse health effects of SSBs by claiming the neglect of confounding factors. Issued statements note that health conditions, such as obesity or diabetes, are complex and caused by numerous contextual factors, which may play an equal or greater role than SSBs. For example, the BSDA commented: “Experts agree worldwide that diabetes is the result of many factors including family history, lifestyle, and weight” in response to a study linking SSBs to diabetes. In another statement, it is claimed that “sugar is not the only thing to blame for rising levels of obesity” and that “the evidence clearly shows that heart disease and other obesity-related diseases such as diabetes are caused by a multitude of factors”.

Notably, the BSDA does not critique observational studies, or those with clear methodological flaws, where results favour its interests. Instead, these studies are quoted by industry. For example, a BSDA press release documents the findings from an industry-funded study by the British Fruit Juice Association with the headline: “Fruit juice drinkers have a lower BMI and waist circumference that non-consumers” [39]. The media release uses the findings to justify the importance of fruit juice consumption despite not being peer-reviewed or published, and its use of secondary data. Moreover, while challenging studies associated with adverse health outcomes for failing to demonstrate causation, the BSDA did not question the same methodology in this study. Based on claims about the shortcomings of observational studies suggesting adverse health outcomes, BSDA spokespersons argue that such findings should not influence policy.

### Industry as an altruistic public health partner

Another strategy used by the BSDA is to position the organization as an altruistic public health partner (*n* = 52). This is pursued in several ways. First, the BSDA publicly acknowledges that health conditions and diseases often associated with SSBs, such as obesity, are significant public health concerns. It then positions the industry as committed to combatting such concerns through industry-led voluntary efforts: “The soft drinks industry recognises it has a role to play in helping to tackle obesity which is why we have led the way in calorie and sugar reduction”. Second, it claims these voluntary industry efforts are the reason for reduced SSB consumption across the UK. For instance, in a 2016 statement on preventing diabetes, the BSDA described how, “In contrast [to a proposal for a SSB levy], the soft drinks industry is taking practical steps to help consumers, through reformulation, smaller portion sizes and increased promotion of low- and no-calorie options - reducing sugar intake by 7.5 percent recent years, and with plans to reduce calories by a further 20 percent by 2020”. Statements put forth with this claim do not acknowledge actions taken by regulatory authorities, public health bodies, academics, or grassroots organizations for this purpose.

### Promoting industry-friendly concepts of safety

The BSDA commonly referenced concepts of safety that align with industry interests to counter research supporting increased SSB regulation (*n* = 47). For example, in a 2015 quote the BSDA director general claimed that “[t] he latest review by the European Food Safety Authority in 2015 confirms that energy drinks are safe and make up a very small part of the caffeine intake of adolescents and a negligible amount amongst children.” Statements cite reputable health and scientific organizations, claiming that these bodies deem their products wholly safe for consumption or when consumed in moderation. For instance, in response to a 2019 study linking SSBs to cancer, the BSDA stated: “The World Cancer Research Fund found no evidence that 100% fruit juice is carcinogenic and maintain that a daily 150ml glass ‘can be part of a healthy, balanced diet and a healthy lifestyle’ and contribute to the 5-a-day fruit and vegetable target”. The BSDA response to the study does not argue that fruit juices are safe but rather that there is insufficient evidence to prove the relationship between fruit juice consumption and cancer. In other words, the BSDA moves from an absolute (‘it is not carcinogenic’) to a conditional statement (“can be part of”) without presenting the conditions that need to be present to make drinking juice ‘healthy’. In this way, the BSDA selectively applies statements from respected bodies, whose policy stance on SSBs are opposed to industry positions, as a source of legitimacy for its claims. For example, the World Cancer Research Fund, which the BSDA cites for fruit juice safety, advocates for SSB taxes, which the BSDA actively opposes [40].

### Focus on unhealthy lifestyles of consumers

Another argument put forth by the BSDA is that unhealthy lifestyles, rather than the consumption of SSBs, should be the focus of public health concern. Healthy lifestyles, in turn, are thus the responsibility of individual consumers rather than industry (*n* = 61). The consumption of SSBs, incorporated into a “balanced diet”, is described as being a part of healthy lifestyles. For example, the BSDA stated that “key risk factors for heart failure include high blood pressure, which is a consequence of an overall unhealthy diet and lack of exercise”. Statements focus on promoting interventions such as health education campaigns, to inform the consumer of health risks, or advocating for healthier lifestyles. For example, the BSDA suggests that “rather than singling out a particular product, we should be encouraging people to enjoy an active lifestyle”. It argues that the development of adverse outcomes is therefore not inherent to a single food or beverage but rather caused by the behaviours of consumers.

## Discussion

Given the fixation by the BSDA - and similar lobbying organizations – regarding the purported lack of evidence of causation between sugar and NCDs, it is imperative that members of the public health community try to educate policy makers about (a) the complex nature of causation; (b) that evidence in favour of public health interventions cannot, and do not, solely rely on causation studies; and (c) that public health must sometimes abide by the precautionary principle in instituting interventions.

To begin, given the social, economic, and political determinants of health, causation is never singular nor linear but rather always multifold and dynamic. Few, if any, biological processes are such that there is only ever one antecedent chemical or process that always causes the same outcome; rather, it is usually the case that a number of antecedents need to be present – each of which might be deemed necessary or sufficient – for a certain outcome to occur. For example, to go from being infected with virus x – which often depends on various environmental factors (e.g., ambient temperature, relative humidity, etc.) - to having the disease associated with virus x depends on numerous factors, including the virulence of the particular strain of the virus and the immune system of the host. The attendant complexity of biology is then extrapolated when we then consider the multitude of social, economic, and political factors that affect the health of individuals and communities. Regarding SSBs, it is true that sugar alone does not cause NCDs, but that sugar plays some important causal role in many NCDs cannot be refuted. On this basis, it is critical that the public health community should counter the BSDA’s claims that sugar does not cause disease by explicitly explaining the role of sugar as a key factor within a complex adaptive system of human health and illness.

Second, it is common practice in public health to institute policies and interventions on the basis of evidence that is not derived from experimental study designs. The hierarchy of evidence that is commonly used in the context of evidence-based medicine (EBM), with the randomized control trial (RCT) and systematic reviews at its apex, does not translate to public health without significant modifications [41, 42]. For example, research questions in epidemiology overwhelmingly cannot be answered using RCTs since study participants cannot and often should not be assigned to pre-determined study arms. As such, public health researchers commonly rely on case-controls, cohorts, or cross-sectional designs. Moreover, given the social dimensions of public health, qualitative projects or surveys are used to derive evidence necessary to answer questions that are relevant to policy makers [43]. This excludes the criticisms that persist against EBM, which critique its purported objectivity and value-free perspectives [44–46]. As noted above, public health must work to inform policy makers about the various sorts of evidence that are used in the discipline that do not rely on establishing causation and experimental studies. In other words, the evidence base around the negative effects of SSB on NCDs is sound without needing to rely on RCTs or causation studies. This fact, and industry claims to the contrary, need to be clarified and reiterated in discussions with policy makers and the broader public.

Finally, even if one were to dismiss arguments about the inherent complexity of establishing causation and the accumulation of evidence that does not rely on experimental studies, public health often invokes the precautionary principle in those instances when the evidence towards a particular conclusion is unclear but the potential negative effects are dire. Although various articulations of the precautionary principle exist, generally speaking, it denotes that when there is uncertainty with regard to a health-related activity or an activity that impacts health, and one can foresee that the activity is associated with significant harm, we ought to err on the side of caution by protecting individuals and populations from the activity until sufficient evidence as to its safety can be ascertained [47, 48]. There are important epistemological and implementation challenges related to the precautionary principle [49]. Yet despite these challenges, the principle is invoked as a means of protecting people from harm in moments of uncertainty. It has been used in public health in a range of instances, from the Rio Declaration on sustainability and the environment [50], to mandating the use of masks and physical distancing in the context of the COVID-19 pandemic despite no definitive proof of how it was transmitted in the early stages of the outbreak. As such, although some uncertainty may persist with regards to SSB and their connection to NCDs, this uncertainty cannot be used prima facie against regulation given the negative effects of NCDs on persons and populations. The precautionary principle, as often used in public health, suggests the opposite: that the onus of evidence lies with SSB industry and lobbyists to provide satisfactory evidence as to its safety.

It is within the above context that we can locate the findings of this paper. In addition to attacking public health research, the BSDA makes three other sustained arguments in its public facing messaging: that it is an altruistic actor, that its products are safe, and that the focus should be on individual responsibility. These arguments are familiar, notably used by the tobacco industry, among other corporate actors [51], for many decades to counter accumulating and substantial public health research on the health harms associated with their products. Tobacco companies have positioned themselves as discouraging youth smoking [52, 53], fought evidence that smoking and second hand smoke causes cancer [54], are not disputing evidence regarding the harms of vaping [55], and tried to focus public health debates on individual choice [56]. The public health community, in turn, has countered these efforts by monitoring and critiquing the industry’s corporate social responsibility claims, taking tobacco companies to court for misrepresenting the harms of their products, and advancing understanding of the commercial, as opposed to behavioral, determinants of health [57]. Perhaps, most crucially, it has convened around an international legal instrument – the Framework Convention on Tobacco Control – which has generated standards for evidence and conflict of interests, and systems for monitoring industry activity and claims [58]. Efforts to country industry influence in debates around SSB can learn from these experiences and emulate these remedies.

At the same time, sugar is not the new tobacco as sometimes argued by public health advocates. The relationship between SSB and poor health outcomes appears more complex, and there are health benefits from the consumption of some sugars in moderation, while consumption of commercial tobacco products has inherently negative effects on health. This means that, as much as public health advocates can and should learn from tobacco control, there is also need for additional strategies. We argue that these could be derived from the precautionary principle, as noted above, including strategies that educate decision-makers and the public about how to evaluate evidence based on public health, not industry, standards which shifts the burden on industry to demonstrate the ‘safety’ of its products.

## Conclusion

In conclusion, the BSDA consistently commented upon SSB research through four arguments: challenging scientific rigour, representing themselves as an altruistic public health partner, promoting concepts of safety aligned with their interests, and shifting focus onto consumer responsibility and lifestyles. The findings demonstrate an important role for public health to educate policymakers and other stakeholders about the complexity of causation, the role of evidence in public health decision making, and the importance of the precautionary principles. This study reinforces the importance of monitoring corporate actors from harm industries and rebutting messages against the interest of public health. Future research is needed to understand, document, and respond to emerging harm-related industries such vaping undermining public health research in public domains.

## Data Availability

The datasets used and/or analysed during the current study are available from the corresponding author on reasonable request.

## Abbreviations

NCDs: Non-communicable diseases
SSBs: Sugar-sweetened beverages
WHO: World Health Organization
ILSI: The International Life Sciences Institute
US: United States
UK: United Kingdom
BSDA: British Soft Drinks Association (BSDA)
